# The Behavioral Intervention Technology Model: An Integrated Conceptual and Technological Framework for eHealth and mHealth Interventions

**DOI:** 10.2196/jmir.3077

**Published:** 2014-06-05

**Authors:** David C Mohr, Stephen M Schueller, Enid Montague, Michelle Nicole Burns, Parisa Rashidi

**Affiliations:** ^1^Center for Behavioral Intervention TechnologiesDepartment of Preventive MedicineNorthwestern UniversityChicago, ILUnited States; ^2^Center for Behavioral Intervention TechnologiesDepartment of Medicine, Division of General Internal MedicineNorthwestern UniversityChicago, ILUnited States; ^3^Department of Biomedical EngineeringUniversity of FloridaGainsville, FLUnited States

**Keywords:** mhealth, ehealth, behavioral intervention technology

## Abstract

A growing number of investigators have commented on the lack of models to inform the design of behavioral intervention technologies (BITs). BITs, which include a subset of mHealth and eHealth interventions, employ a broad range of technologies, such as mobile phones, the Web, and sensors, to support users in changing behaviors and cognitions related to health, mental health, and wellness. We propose a model that conceptually defines BITs, from the clinical aim to the technological delivery framework. The BIT model defines both the conceptual and technological architecture of a BIT. Conceptually, a BIT model should answer the questions why, what, how (conceptual and technical), and when. While BITs generally have a larger treatment goal, such goals generally consist of smaller intervention aims (the "why") such as promotion or reduction of specific behaviors, and behavior change strategies (the conceptual "how"), such as education, goal setting, and monitoring. Behavior change strategies are instantiated with specific intervention components or “elements” (the "what"). The characteristics of intervention elements may be further defined or modified (the technical "how") to meet the needs, capabilities, and preferences of a user. Finally, many BITs require specification of a workflow that defines when an intervention component will be delivered. The BIT model includes a technological framework (BIT-Tech) that can integrate and implement the intervention elements, characteristics, and workflow to deliver the entire BIT to users over time. This implementation may be either predefined or include adaptive systems that can tailor the intervention based on data from the user and the user’s environment. The BIT model provides a step towards formalizing the translation of developer aims into intervention components, larger treatments, and methods of delivery in a manner that supports research and communication between investigators on how to design, develop, and deploy BITs.

## Background and Purpose

A growing number of investigators have commented on the lack of models informing the design of behavioral intervention technologies (BITs) [1]. We use the term BITs to refer to behavioral and psychological interventions that use a broad range of technologies, such as mobile phones, the Web, and sensors, aimed at changing behaviors and cognitions related to health, mental health, and wellness [2]. To date, some BITs use psychological models, such as the Theory of Planned Behavior or Social Cognitive Theory, to inform design [3]. While these models are useful in describing the types of behavioral and cognitive changes required of users in their environments to achieve clinical targets, they offer little information on how to design and implement BITs to ensure that they are useful and usable [1]. Specifically, these models are limited in at least two ways. First, they focus on clinical outcomes, which are often more distal to the behavioral targets of specific BIT interventions and are unlikely to change within the timeframe necessary to inform design considerations or to submit BITs to rapid development, evaluation, and iteration [4]. Second, purely psychological models do not include critically important factors that can guide the design and specifications for a BIT.

The purpose of this paper is to describe a BIT model that supports the translation of the clinical aims of a BIT treatment and its intervention components into BIT features. The BIT model proposed here is intended to provide a broad hybrid framework that combines behavioral principles with technological features that can help bridge the fields of behavioral science and technology. Experts from both fields contribute to the development of BITs, but the vastly different training and knowledge backgrounds have led to differences in conceptual models that guide development and evaluation. A framework that integrates behavioral science, design, and engineering can support the definition of systems in terms of testable hypotheses that could then be evaluated. This would help avoid the all-too-common process of developing BITs that ignore psychological or engineering principles or that rely entirely on developer intuition [1,3].

## Review of Existing Models

### Overview

We review here three design models proposed by Ritterband [5], Fogg [6], and Oinas-Kukkonen [7,8] that have informed the development of BITs. This review is intended to provide a context for the proposed BIT model and not as an exhaustive review and critique of theoretical models used to inform the development of BITs. Other sources provide a more exhaustive review of the different models of behavior change used to inform BITs development [3,9].

### Ritterband

Ritterband provided one of the first generalizable models depicting how a Web-based intervention contributes to symptom change [5]. The model posits that website design, human support (eg, a coach or therapists), user characteristics, and environmental factors contribute to website use, which in turn leads to behavior change and ultimately symptom improvement. The model also describes, although does not categorize, attributes of a website including intervention components (eg, media, messaging, assessment) and quality attributes (eg, appearance, difficulty of use, accuracy of information) intended to support behavior change.

The Ritterband model is useful, as it specifies the elements and characteristics to consider when designing an intervention website. Many elements of the model could also be applicable to other technologies, such as mobile devices. However, the Ritterband model does not articulate how technological components might be mapped onto more specific (and proximal) intervention goals, which is important in intervention design. Furthermore, while Ritterband emphasizes that the model is not necessarily linear (eg, components do not necessarily need to be deployed sequentially), the non-linear properties are not articulated. These non-linear properties are increasingly important as technologies are able to receive and react to data obtained from the user, the user’s environment, and third parties such as a health care system or coaches.

### Fogg Behavioral Model

The Fogg Behavior Model [6] is a model for understanding behavior change that identifies the factors that control whether a behavior is performed. As it focuses on specific behaviors, it is most applicable to changing small, clearly defined behaviors, which he refers to as “tiny habits”. The model does not purport to guide applications focused on changing attitudes or cognitions nor does it guide applications that target more complex treatment goals. Fogg focuses on three constructs: motivation, ability, and triggers. Motivation and ability are inversely related such that simpler behaviors require lower levels of motivation to initiate. Triggers are events in the environment (or from an intervention) that elicit the behavior at a given level of motivation. Fogg does not believe that technologies are particularly effective at teaching new behaviors, rather, he argues that they are best suited to simplifying tasks, thereby increasing ability and providing triggers that might initiate the desired behavior when applied at the appropriate time (ie, when appropriate given the level of ability and motivation). He argues that the best design of BITs is to be responsive to an individual’s motivation and to adapt the behavior (through simplification) or the environment (through triggers) appropriately. A key requirement of Fogg’s model is that the initial target behavior be small; larger behavioral goals can be achieved through the concatenation of smaller goals.

Fogg’s model is elegantly simple and very useful within the constraints he outlines. However, the restricted focus does not fit the goals of many treatment interventions that attempt to address more complex problems such as reducing symptoms of depression or anxiety, treating insomnia, improving self-management of chronic illnesses, coping with addictions, or implementing healthy lifestyle programs. Users may not know what steps to take to attain their goals and may require some education. It may even be difficult for users to identify behavioral goals that are circumscribed enough to be attainable. Motivation may wax and wane and thus can be a focus of BITs. However, Fogg’s model may be very useful for small behaviors. As such, the model could serve as a useful tool in considering and designing individual components of larger intervention programs.

### Persuasive System Design

Oinas-Kukkonan has described comprehensive models, which he refers to as Persuasive System Design and the Behavior Change Support System, which, in spite of the name, also addresses cognitive change and adherence to a BIT [7,8]. Oinas-Kukkonen identifies 3 areas of change: (1) forming a behavior, cognition, or BIT adherence behavior, (2) altering a behavior, cognition, or BIT adherence behavior, and (3) maintaining a behavior, cognition, or BIT adherence behavior. To this we would add stopping or reducing a behavior or cognition, such as unhealthy eating or addictions. Oinas-Kukkonen also identifies four general design features, each of which contains a number of more specific components: (1) primary task support, which includes reducing complex behaviors into simpler ones, tunneling experience, tailoring and personalization, self-monitoring, simulation, and rehearsal, (2) dialogue support, including positive reinforcement, reminders, and suggestions, (3) credibility, by conveying trustworthiness and expertise, and (4) social support, including both social networking components and the provision of social normative information.

A strength of Oinas-Kukkonen’s model is that it supports the transfer of design components into software functionality. Its clear articulation also allows the evaluation of the value of these components, as evidenced by a meta-analysis that evaluated both the frequency of the use of these components, as well as their impact on adherence [10]. While this model links intervention aims with a variety of well-articulated intervention elements, it does not discuss how individual intervention elements may be varied or integrated into a larger treatment program.

## BIT Model Description

The BIT model provides a framework for the translation of treatment and intervention aims into an implementable treatment model. For the purposes of clarity, we use the term “intervention” to refer to a single interaction with a single element and the term “treatment” to refer to multiple interactions that unfold over the entire course of interaction with the BIT.

**Figure 1:**
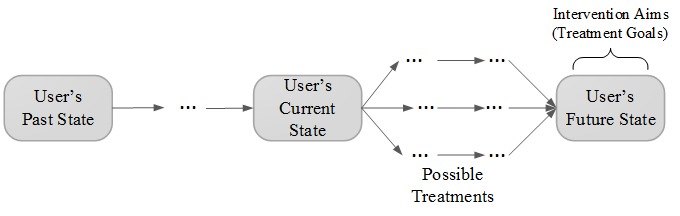
BITs facilitate reaching future changes (ie, intervention aims) through possible interventions.

BITs are intended to assist users in achieving a goal related to health, mental health, or wellness. A single BIT intervention enables users to change their current state (the state at the moment of the BIT use) using one or more possible interventions to achieve the intervention aims (desired future states) (see Figure 1). We use the term “Past State” to indicate prior states and events. This time point can be defined depending on the application context (eg, events in the past hour, or before yesterday). The future state can be defined in a similar manner. A BIT treatment can be defined as a concatenation of these BIT interventions over time.

The BIT model displayed in Table 1 displays the “why”, “what”, “how (both conceptual and technical)”, and “when” of BITs. The theoretical level consists of the “why” and conceptual “how”, whereas the instantiation level consists of the “what”, technical “how”, and “when”. Most BITs consist of a sequence of intervention steps delivered to the user, each intended to achieve a specific *aim* related to the broader treatment goal, such as monitoring calorie intake for a weight-loss intervention. The aim in Table 1 describes the *why* of any specific intervention and intervention component and reflects the intention of a developer. *How* an aim is achieved is defined by a behavior change strategy, which conceptually defines more proximal aims that support the user in attaining an aim. Each behavior change strategy is instantiated by a BIT element or set of elements, which are more granularly defined intervention components of the overall BIT treatment. Elements are the *what* of the model. *How* an element is displayed is affected by the characteristics, such as the output complexity (difficult, easy) or the medium (video, text, etc). This *how* refers to the technical rather than conceptual considerations. Because a BIT is considered to be a sequence of intervention elements delivered to the user over time, the relative order and rules for progressing through the BIT must be defined. The workflow describes *when* each intervention element will be displayed by determining the precedence of features and conditions under which intervention elements may be delivered. In the following sections, we discuss each of these components in more detail. The overall BIT model consists of these 4 constituent parts (aims, elements, characteristics, and workflow). As “interventions” refer to individual instances or interactions with the user, it includes the elements and their characteristics. “Treatment” refers to how these aspects unfold over time and thus adds workflow.

**Table 1:** Summary of BIT model.

		BIT component	Examples		
**Theoretical**					
	Why	Aims	Clinical aims:		
				Weight reduction:	
					Decrease caloric intake
					Increase physical activity
				Promote sleep hygiene	
				Decrease depression:	
					Increase positive activities
					Decrease avoidance behaviors
			Usage aims:		
				Use of Intervention tools	
	How (Conceptual)	Behavior change strategies	Education		
			Goal setting		
			Monitoring		
			Feedback		
			Motivation enhancement		
**Instantiation**					
	What	Elements	Information delivery		
			Notifications		
			Logs		
			Passive data collection		
			Messaging		
			Reports		
	How (Technical)	Characteristics	Medium		
			Complexity		
			Aesthetics		
	When	Workflow	User defined		
			Frequency		
			Conditions:		
				Time-based rules	
				Task completion rules	
				Event-based rules	
			Tunneling		

### “Why”: Intervention Aims

The overall goals of a BIT treatment, as well as the aims of any given intervention component, reflect the intentions of the developer [8]. In the context of BITs, these aims can generally be classified into two somewhat overlapping classes: clinical and usage aims. Clinical aims refer to changes in behaviors, cognitions, knowledge, skills, and motivation for treatment-related behaviors. Clinical aims refer to the clinical goals of the intervention or treatment (Table 1 shows examples such as increasing weight reduction, promoting sleep hygiene, or decreasing depression). Often the larger treatment goal also includes hierarchies of sub-aims, each of which supports the attainment of the larger treatment goal. For example, to decrease depression, sub-aims may be to increase positive activities that bring pleasure or a sense of accomplishment, as well as reducing avoidance behaviors that prevent the individual from engaging more fully in life [11]. Similarly, a weight reduction aim may include decreasing caloric intake and increasing physical activity. The hierarchy of aims should be as specific and as clearly defined as possible to facilitate a clear treatment plan.

Usage aims focus on maintaining engagement with the BIT generally and/or with its specific intervention components. Usage aims are often thought to be related to clinical aims, although the relationship between use and outcome has been mixed [12]. Furthermore, in many studies, investigators examine usage outcomes as a proxy for proximal outcomes, although we would caution against that as these are better treated as distinct rather than interchangeable concepts. Usage aims, are more frequently employed in studies conducted by technologists, such as usability testing. These studies use theories, such as the technology acceptance model, that imply that the perceived usefulness and ease of use of the intervention contribute to an individual’s motivation to continue using the application [13,14], while psychologists and behavioral scientists tend to focus more on clinical outcomes.

### “How” (conceptual): Behavioral Intervention Strategies

Behavioral change strategies are the methods used to attain clinical and use aims. They are grounded in models and theories of how behavior change occurs and is maintained. Table 1 provides examples of common behavior change strategies, which are described below. This list is drawn from Michie’s extensive taxonomy of behavior change strategies [15,16] and is not intended as a comprehensive list. Many of these strategies can relate to either clinical or usage outcomes. For example, education may focus on providing the requisite knowledge to change a behavior related to a clinical aim and/or provide instruction on why and how to use the application to increase its usage. A critical step, however, in selecting which aims to promote is to have a clear rationale for how a given strategy will support the overall goal of treatment.

Education aims to increase the user’s understanding of their past and current state and of the steps required to achieve the future state (see Figure 1). Examples include providing information and instruction and may include material about the problem, treatment rationalization, information on consequences of behaviors, modeling, and demonstrating a behavior. In addition, education may include instruction on how to use the application.

Goal setting involves future planning to achieve desired future states. This can include activity scheduling, setting tasks of progressively greater difficulty, anticipation of barriers, or goals with respect to application use.

Monitoring involves recording of past states or current states. Examples include recording of current or past behaviors, cognitions, or events, reviewing previously set goals and identifying barriers, or monitoring intervention and application use.

Feedback provides information on current and past states, or the likelihood of future states, with the goal of increasing insight and understanding regarding the user’s condition or actions. Feedback may also overlap with other behavior intervention components, such as motivation enhancement (eg, feedback on goal attainment may provide information about progress and may also increase or decrease motivation).

Motivation enhancements are interventions that increase the likelihood that the user will engage in specific behaviors related to treatment goals or use of the application in the future (motivation to change current state into future state through behavior change or BIT use). These include positive reinforcement, contingent rewards, behavioral contracts, incentives, and social support [10]. Providing opportunities for social comparison and identification with role models are examples of motivational enhancement that utilize social support elements.

### “What”: BIT Elements

BIT elements are distinct components or objects of a BIT intended to implement the behavior change strategies, which in turn support the user in achieving the clinical and usage aims required to attain the treatment goal. By BIT elements, we mean the actual technical instantiations present in the BIT. For example, a data entry field created in a food logging application supports the behavior change strategy of monitoring. Thus, the BIT elements are the aspects of the BIT, with which the user actually interacts. Below is a list of commonly used elements of existing BITs, but this list could expand as aims, designs, and technologies continue to advance.

Information delivery typically involves one-way interactions in which the system provides content to the user when the user initiates access. These can include such things as text, video, images, audio, or a combination of media. They are distinct from other similar components in that they commonly remain available after release and are often used didactically.

Notifications are individual messages pushed to the user, such as text messages, emails, or within app notifications.

Logs are a form of data collection that require the user to enter data. Examples include free entry, selection menus, and using a rating scale.

Passive data collection refers to data collected without any user effort, such as phone sensor data collection, data from external devices such as pedometer, and data collected through application programming interfaces (APIs) from other available sources (eg, weather data or prescription refills).

Messaging elements link the user with other individuals including those supporting the interventions (both professionals and paraprofessionals), peers drawn from their social network, or peers using the system. Messaging refers to more than just one-to-one correspondence and can include discussion boards.

Reports are reflections of data collected by the BIT that are provided back to the user (eg, calendars, calorie counts, thought records).

Visualizations may be considered a subset of reports and convey specific information derived from previously collected data and assessments. Data may be aggregated across an individual user or across groups of users.

BIT element(s) are mapped onto behavior change strategies. A specific behavior change strategy can be targeted by more than one BIT element, which may be delivered sequentially or may be embedded in each other. For example, education is often achieved by delivering didactic tools that rely on text-based information. But such learning may be augmented by embedded reports (visualizations or text) derived from data and assessment, thereby providing feedback to illustrate a point and support learning.

### “How” (technical): Characteristics

BIT elements can be further defined and/or refined across a number of dimensions to better fit the user and/or optimize the element to achieve its aim and overall treatment goal of the BIT, commonly by improving the user’s comprehension, ability to complete tasks, and engagement. We describe four characteristics (medium, complexity, aesthetics, personalization) that have received attention in BIT research, however, these are intended as examples and are by no means an exhaustive list.

Medium refers to media employed, such as text, video, audio. Variation in the medium can be varied for many of the intervention elements, including information delivery, social networking, or data collection. In considering the medium, it can be useful to apply a framework, such as media richness theory, which can provide information on how media may vary in their suitability for communicating different types of information effectively [17]. The media richness hierarchy is organized from high to low levels of richness based on the capacity of media types to process information or cues. Each cue can be assessed on multiple criteria including (1) speed of feedback (fast, slow, instant), (2) the capacity of the medium to transmit multiple cues simultaneously, (3) the ability to use natural language, and (4) the personal focus of the medium. Richer media are not necessarily better [18]; the aims of any given intervention element are most likely to be effective if the communication channel fits the task and the capabilities of the users [19]. For example, video may be a better media for communicating information to users who have low literacy, while text may be preferable to more educated groups.

Complexity can be varied depending on the user, target population, and the task (eg, providing didactic information, a notification, or data collection). For example, some users may prefer more elaborate content, while others may prefer leaner content [20]. Or, for logging features, some users prefer the control and specificity afforded by free text entry, while others prefer the simplicity of drop-down menus. The complexity of content or tasks may vary by user capabilities and limitations such as educational level or familiarity with device. The complexity of content and tasks may also vary based on the context in which application is used (eg, at home, work, or in transit).

Aesthetics may vary depending on the user characteristics and tastes [21]. Aesthetics can have a substantial impact on user acceptance and usability [22]. There are engineering principles of aesthetics that relate to user acceptance and performance that should be considered [23].

Personalization refers to altering the characteristics or content of a BIT to increase the relevance for an individual user. For example, the content of information may be tailored to fit the user’s needs and capabilities by altering language or providing examples that are more likely to be relevant to the user [24]. Personalization has generally relied on predetermined criteria to adapt the form of interventions; however, it is also possible to use machine learning methods that can learn from population and individual user data to automatically adapt the form of interventions to meet the user’s needs and capabilities [25-27]. Personalization can impact the characteristics of the medium, complexity, and aesthetics, where these characteristics are made more relevant based on individual user needs and characteristics.

The characteristics in this model are intended to reflect the need to modify BIT elements. Conceptually, the elements could be considered objects, and the characteristics could be considered the potential attributes of those objects. It is beyond the scope of this paper to provide guidance on the methods one might use to decide which attributes best meet the needs of users, as these questions are the subject of entire fields of study such as human factors engineering and human computer interaction (HCI).

### “When”: Workflow

Most BITs are designed for repeated interactions over an extended period of time. That is, within our terminology, most BITs are intended as a treatment consisting of a series of interventions. The workflow defines when and under what conditions BIT interventions are delivered and can take into account changes in the aims, elements, and/or characteristics that occur over the course of a treatment. The workflow identifies when an intervention is delivered and potentially also the sequence of interventions. Below we describe common examples of workflows (user defined, frequency, conditions, tunneling).

User defined workflows allow the user access to all intervention elements and content, permitting the user to decide the sequence and timing of their use.

Frequency refers to the frequency with which any intervention is deployed. Some interventions have expectations of the frequency of use.

Conditions use data to determine when an intervention will be delivered. A variety of types of conditions can be employed. (1) Time-based rules define the release of an intervention element based on the passage of time. For example, Web-based treatments modeled on standard face-to-face treatments sometimes release new content on a weekly basis [28]. (2) Task-completion rules define the release of intervention elements based on the user’s completion of prescribed intervention tasks, such as the completion of a pre-determined number or set of activities detected by the intervention system. (3) Event-based rules define the release of elements based on the detection of criteria detected by the intervention. Events may be derived from user-entered data (eg, a patient characteristic or change in state), sensor data, or any other data (eg, data from an electronic medical record). An “event” may also be defined as the absence of data (eg, a notification may be provided when no user activity has been detected over a given period of time).

Tunneling uses data to determine which interventions are most like to meet the needs or preferences of an individual at a given time. For example, an intervention for anxiety can use information on comorbidities to provide specific interventions targeting those problems to improve efficacy [29]. As with personalization, adaptive systems, using artificial intelligence and machine learning techniques, can potentially use population-level data along with individual user data to determine the workflow of an intervention, similar to commercial recommendation systems such as Netflix or Amazon [25-27].

Workflows may use and integrate a number of these elements, for example, providing core interventions in a predetermined sequence with a mixture of time-based and task completion rules and then allowing the user to select from a variety of additional interventions that the user believes are most useful [30,31].

## BIT Model: Example Using MyFitnessPal

To further explain the BIT model, we provide an example of a portion of a popular fitness app (MyFitnessPal). MyFitnessPal is an Internet website and mobile application designed to help people lose weight. The MyFitnessPal mobile application is freely available for the Android, BlackBerry, iOS, and Windows platforms. The overall clinical aim of MyFitnessPal is to promote weight loss. Two of the sub-aims of the application are to reduce caloric intake and increase physical activity. Although MyFitnessPal makes use of several behavior change strategies (education, feedback, goal setting, motivation enhancement), the major behavior change principle used is monitoring. That is, weight loss is promoted by helping people track what they eat and how much they exercise. Reviewing every feature of MyFitnessPal is beyond the scope of this paper; however, we present aspects of the core functionality of entering food into one’s diary to illustrate the BIT model.

**Figure 2:**
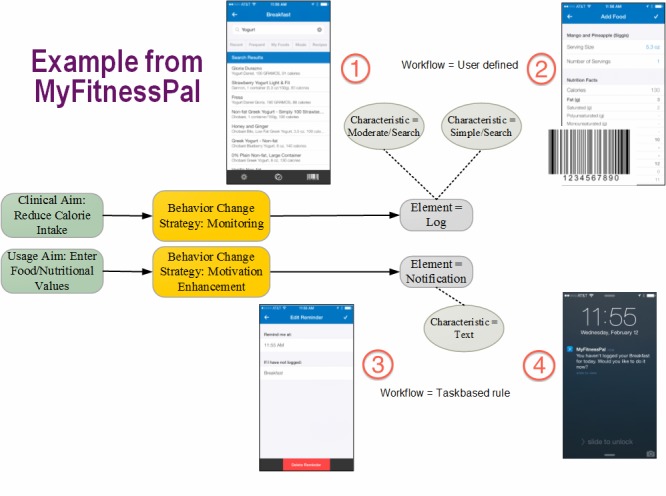
BIT model example using MyFitnessPal calorie intake monitoring features.

Figure 2 displays the BIT model as it applies to a single action, that is, logging one’s breakfast into the MyFitnessPal diary. Starting with the clinical aim of reducing caloric intake, the behavior change strategy is monitoring one’s food intake. Thus, the behavior change strategy (monitoring) bridges the clinical aim (reduction of caloric intake) and the technological instantiation. The BIT element with which a user initiates the interaction with the application is a food log. The food logging element has a number of potential characteristics (how). A user can enter the nutritional data in free text. To simplify this entry, a user can search for a particular food, in this example yogurt, in order to input that specific nutritional data into one’s diary (see element 1 in Figure 2). However, an even simpler form of entry allows the user to scan the barcode of the food item (see element 2 in Figure 2). MyFitnessPal will then use the barcode to search for the item, eliminating the need for the user to search and then correctly select the item from the items within MyFitnessPal’s database. The workflow here is user defined.

The use of the food diary requires the user to initiate the interaction, requiring the user to remember and be sufficiently motivated to engage in the task. To mitigate the effects of forgetfulness or low motivation, MyFitnessPal makes use of another behavior change strategy, motivational enhancement, to support the usage aim of entering consumed food into the application. One technological manifestation of a motivation enhancer is through the BIT element of a notification. In MyFitnessPal, these notifications are delivered via text push notifications from the application. These notifications are created from the system using task completion rules. As Figure 2 illustrates, the notification is programmed into the system to be delivered at a specific time if, and only if, the food logging task has not been accomplished (see element 3 in Figure 2). In this example, a notification to log one’s breakfast is provided if breakfast has not been logged by 11:55 a.m. (see element 4 in Figure 2). The characteristic of the notification is text (as opposed to audio or visual). In this way, a user can forgo receiving the notification by engaging in the user-defined workflow prior to the programmed event that will initiate the task completion rule leading to the BIT element of the notification.

The MyFitnessPal example is an illustration of how the BIT model maps onto an existing application. High quality applications often contain these elements; however, there is no shortage of poorly designed applications available that do not effectively engage users to accomplish the intended actions or achieve the intended aims. The BIT model is intended to support developers and designers by providing a clear model of how to move from a general clinical aim to a clearly defined and effective application. We now move to a discussion of translating these conceptual design decisions into technological implementations.

## From Theory to Application: Technological Implementation of the Model

The instantiation of a design based on the BIT model requires technological implementation in a system that can actually deliver the BIT to users. In this section, we provide an example of a hybrid model that integrates a general technological framework using the BIT model. We refer to this as BIT Technological, or BIT-Tech. BIT-Tech is an example that can be used by system designers and developers as a conceptual guideline. It shows the relationship among (1) software components developed for supporting BITs, (2) the user, and (3) the environment.

BIT-Tech is defined with respect to the previously defined BIT model concepts, that is, intervention *(I)*, aim *(A)*, element *(E)*, characteristics *(C)*, and workflow *(W)*. Data, denoted by *D*, can be acquired from a variety of sources. We denote user data by *D(U)* and environmental data by *D(E)*. User data include data related to user, such as demographic information, activity data collected by an accelerometer, and entered calories. Environmental data include data from environment (and not the user). Examples include weather data, traffic data, and geo-location information. Again, each intervention *(I)* is a combination of an element and its characteristics I=<E, C>. The benefit of BIT-Tech is that it is not a separate model, but rather an instantiation of the BIT model in technological form. Thus, both aspects of the BIT model (those displayed in Table 1 and the BIT-Tech displayed in Figure 1) are equally important for the model.

We use the superscript notation *A* ^*t*^ to refer to the specific *aim* at time *t*. This time step will be defined by the system designers depending on the treatment’s needs and may represent a precise moment, for example, a second or a longer block of time, such as an hour, a day, or a week. The same notation is used for other concepts at time *t*: intervention component *(I* ^*t*^ *)*, intervention element *(E* ^*t*^ *)*, characteristics *(C* ^*t*^ *)*, workflow *(W* ^*t*^ *)*, and data *(D* ^*t*^ *)*. Note that if the system is stationary, that is, it does not change over time, we can simply eliminate the superscripts. It also should be noted that the units for *t* selected may correspond to the application context, computational resources, need for a fine-grained versus coarse-grained intervention, and/or the specific aspect of the BIT model. For example, one might represent *t* in terms of seconds for aspects such as data to create a “just-in-time” intervention, but in terms of weeks for aspects, such as workflow if the designer wants the conditions that trigger interventions to be consistent for the length of the intervention.

**Figure 3:**
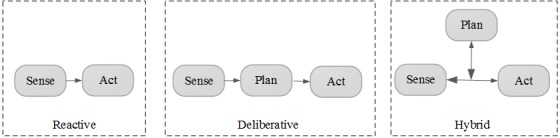
Three paradigms: Reactive, Deliberative, and Hybrid.

Inspired by the robotics paradigm, we will describe our model in terms of *sensing*, *planning*, and *acting* primitives. As described in the literature, in a reactive paradigm (see Figure 3), there are multiple instances of *sense-act* coupling, where each instance processes the sensed data independently and acts independently [32,33]. In a deliberative paradigm, data are sensed from different sensing modules and integrated into a global model, then an action is planned, and next the action is executed. Finally, the hybrid model is a combination of both paradigms, where a direct *sense-act* coupling exists, but the data can also be used by the planning module. The latter allows for inclusion of a planning component, while also providing the flexibility of the reactive models such as layered architectures [34].

**Figure 4:**
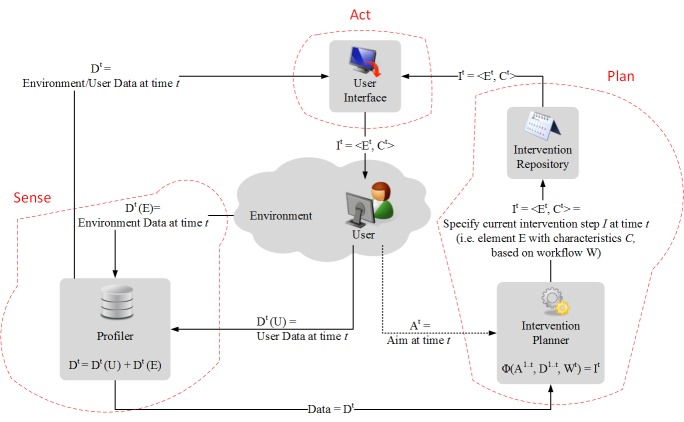
BIT-Tech framework: required environment and user data is collected by the Profiler component; collected data is passed to the Intervention Planner, which is responsible for planning intervention at time t; the Intervention Repository component stores all the interventions and passes specific details of the selected intervention to the User interface component, which then delivers the intervention.

The BIT-Tech aspect of the model is composed of several components (Figure 4):

- Profiler: The profiler is responsible for collecting data to define the user and environment at any given point in time. The profiler passes data *(D)* to the intervention-planner component. This corresponds to the sensing module in the hybrid paradigm.
- Intervention Planner: The intervention-planner is responsible for planning interventions at current time *t* by choosing the relevant intervention elements *(E)* with characteristics *(C)*. Exact mapping of intervention (as defined in Equation 1) will depend on the application needs and will be determined by the developer at design time.
- Intervention Repository: The intervention repository stores all the intervention elements developed for the use with the BIT and can be implemented in terms of a database. Once the intervention repository receives the specification of the current intervention step from the intervention-planner at time *t*, the specification will be passed to the “User Interface” component. The Intervention Planner and the Intervention Repository components together comprise the planning module in the hybrid paradigm.
- User Interface: This delivers an intervention *(E+C)* using a user-friendly interface. The user interface corresponds to the acting module in the hybrid paradigm.

The unfolding of these interventions over time is specified by the workflow *(W)* and influenced by the available data *(D)*. As workflow aspects are considered, specific interventions *(I)* combine to create larger treatments intended to achieve the clinical goals.

Note that the selection of aims and elements can be predefined by the BIT based on the developer’s expertise, or alternatively can be chosen by the user or may be determined adaptively based on information received during the intervention. A treatment as a sequence of intervention steps is defined in terms: (1) elements *(E)*, (2) characteristics *(C)*, and (3) relative order and transition condition of intervention steps, all determined according to workflow *(W)*. Thus, the intervention-planner’s function *Φ* can be defined according to Equation 1. It uses aims *A*, data *D,* as well as workflow *W,* as the input and provides an intervention step specification *I*=< *E* ^*t*^,*C* ^*t*^ > is the output. The intervention-planner function *Φ* typically will be designed based on designer’s definition of the workflow to determine the transition between intervention steps. Therefore, Equation 1 is as follows: *Φ* (*A* ^*1..t*^ *,* ^*D1..t*^ *,W* ^*t*^) = *I* ^*t*^= <*E* ^*t*^ *,C* ^*t*^>

More specifically, the workflow *W* is defined in terms of a finite state machine [35]. A finite state machine (FSM) is a graph used in computer science as an abstract model of programs. Each FSM has a finite number of states (graph nodes), and the machine is at one of the states at any given moment (called current state). It can change from current state to another through graph edges (called a transition) when a triggering event happens or a condition is satisfied. In our model, the states represent the intervention steps, and the intervention steps proceed through transitions (see Figure 6 for an example workflow).

**Figure 5:**
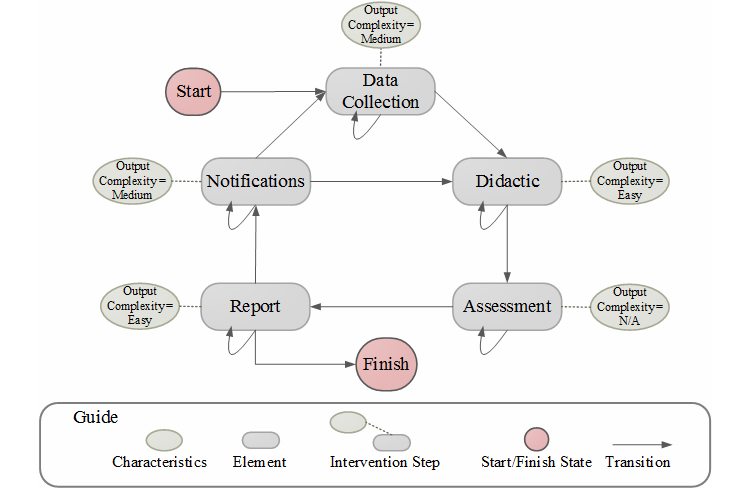
Example workflow generated by the workflow-planner specifying the elements (rectangular nodes), element’s characteristics (elliptical nodes), as well as order of transitions among elements.

The transition among intervention steps is defined by function *Φ*. In general, a transition depends on the previous intervention steps according to the workflow, as well as previous aims, historical data, and current time, as in Equation 1. It should be noted that depending on the specific needs of the system and the available computational resources, one might store/use all the historical information or use only the most recent data. Note that there also may be self-transitions. For example, if the user does not respond to a notification, then the notification can be repeated (a self-loop). In Figure 5, all intervention steps have self-transitions. The self-transitions determine the frequency of a specific intervention step (ie, how many times it will be repeated). Figure 5 shows another example of a workflow based on our MyFitnessPal.

The transition function might also be designed using partial contextual information. For example, a transition might be triggered simply if a certain amount of time has elapsed (eg, 1 week) or by the completion of specified tasks, regardless of the contextual information about previous interventions, aims, and collected data. However, it is also possible to begin developing adaptive BITs that employ artificial intelligence techniques to adapt the workflow to the user’s preferences and/or needs over time. That is, the workflow structure can be modified over time using collective and individual data to provide and sequence specific intervention elements with specific modifications to the characteristics to increase the likelihood of achieving the treatment and intervention aims.

Data gathered through the profiler may be initialized with specific profile data, such as demographic information and clinical status (user data), or specific time and location (environment data), which may determine the BIT tools delivered, any tailoring or refinement of the elements, characteristics, and workflow. The profiler may also gather additional types of data over the course of the treatment, such as updated data on clinical status, information on the patient’s use of the application elements, or environmental data such as location or weather (see Figure 4).

After the system is developed and deployed, the system performance and effectiveness can be evaluated using different computational metrics. For example, the interaction aspects of the BIT can be evaluated using HCI measures such as usability, ease of use, and usefulness [36,37]. Other aspects of the system related to software quality, such as reliability, security, maintainability, and efficiency, can be evaluated using software engineering metrics [38-40]. Finally, if the system is using artificial intelligence and machine learning techniques, related metrics such as accuracy and precision of predictions and recommendations can be used [41,42].

**Figure 6:**
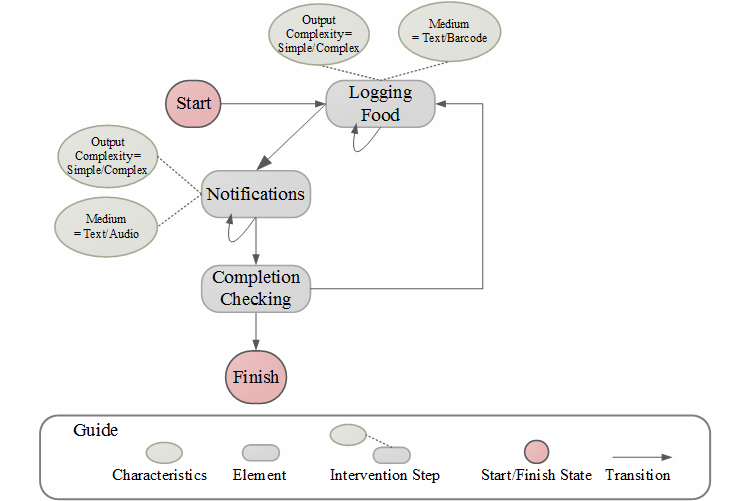
Workflow for MyFitnessPal specifying the elements (rectangular nodes), element’s characteristics (elliptical nodes), as well as order of transitions among elements.

## Discussion

### Implications

We have described the BIT model, which includes both a framework for articulating the relationship between intervention aims, elements, characteristics, and workflow and its technological counterpart (BIT-Tech). This model has a number of potential uses and implications for BIT research.

The BIT model can help developers formalize their intentions with respect to each design consideration, as well as assist in the clarification of how the intervention aims will be implemented in terms of intervention elements. This formalization can be assisted through the development of checklists and flow diagrams that allow the developer to utilize the general model in the development of a specific intervention. In this way, the BIT model can guide development (particularly those who are new to the area) to think through the various decisions that are critical to the design of a BIT and promote the integration of behavioral and psychological theory with BIT design.

Much of the development of BITs to date has been informed primarily through the application of behavioral and psychological theories and by developer intuition [1]. Behavioral and psychological theories provide guidance regarding the relationship between user behaviors and the attainment of ultimate treatment goals and may be helpful in determining proximal intervention aims, but they are less helpful in guiding the development of elements, characteristics, and workflow. While developer intuition has contributed to the rapid growth of BIT research, the use of clear models that formalize and make developers’ intentions transparent will facilitate the communication of those intentions. Clear communication about design intentions will support the exchange of ideas and the growth of the field.

The formalization of the design and development process also supports the translation of the developer’s intentions into testable hypotheses regarding the effects of specific intervention elements, characteristics, and workflow decisions, as well as determining the required data outputs to test those hypotheses. Much of the evaluation of BITs has focused on efficacy, which limits the growth in our knowledge regarding the mechanisms by which BITs achieve both their proximal intervention aims as well as the ultimate treatment goals [4]. Attaining some level of consistency in how aims, elements, characteristics, and workflow are defined would facilitate the evaluation of these components across studies [10].

The proposed conceptual framework could be further refined through the development of more detailed ontologies that further define BIT elements, characteristics, and workflows. An ontology is a formal language used to create a map of a domain, which can provide the conceptual framework to facilitate the rapid or automated construction of BIT applications [43]. Ontologies can also allow data to be defined consistently, allowing it to be queried and retrieved in structured ways [44]. A well-defined generally accepted ontology would facilitate interchange of information across diverse systems by describing the data at various levels of detail, independent of the particular names used in any one system [43]. This would facilitate investigations across treatment protocols and potentially across research groups.

### Limitations

There are several caveats and limitations of the present work that should be mentioned. First, the BIT model we present is intended to be generalizable and is therefore a simplification. It is intended as a general framework that should be modified and elaborated to fit the needs of a specific BIT treatment protocol. Second, the proposed model is intended to respond to calls for a framework that integrates developers’ intentions, behavioral and psychological theory, the design of BIT treatment protocols, and the implementation in a technology framework. We fully expect and encourage the modification of this framework to take into account the ideas of other investigators, new technological developments, the needs and intentions of other stakeholders such as purveyors and care systems [45,46], and most importantly, the development of data that can be used to modify and refine the model. In short, this is intended as a starting place for the development of more comprehensive models and theories that can guide development and research in BITs. Finally, the BIT model has not integrated design processes, such as user-centered design. This model is intended as a high level model that can be used as the basis to develop an ontology, and not to guide specific instantiations of a BIT. Design processes that integrate information on the needs, desires, and limitations of users into the development process are also critical to ensuring that BITs are usable and useful [47].

### Conclusion

The BIT model builds on existing models. Our BIT model extends the Ritterband model [5] by including the intentions of the developer and by increasing the level of granularity. The Fogg Behavioral Model [6] can be used to explain a user’s engagement with any specific intervention element, or set of elements. The BIT model extends the work of Oinas-Kukkonen [7,8] by allowing more granular definition of elements, characteristics, and workflow. The BIT model provides a step towards formalizing a map that can translate clinical aims into behavioral strategies, application specifications, and delivery systems in a manner that supports design, the development of testable hypotheses aimed at improving BIT design, and communication between investigators and across research groups.

## Abbreviations

API: application programming interface
BIT: behavioral intervention technology
BIT-Tech: Behavioral Intervention Technology—Technological Instantiation
FSM: finite state machine
HCI: human computer interaction
